# Machine Learning Models for Predicting Bleeding Risk in Anticoagulated Patients with Atrial Fibrillation and Venous Thromboembolism: A Comparative Evidence Synthesis

**DOI:** 10.3390/jcm15062370

**Published:** 2026-03-20

**Authors:** Winnie Z. Y. Teo, Maggie Wing Yin Wong, Fang Jin Lim, Emmeliene Su-Min Ong, Nesaretnam Barr Kumarakulasinghe, Eng Soo Yap

**Affiliations:** 1 Department of Haematology-Oncology, National University Cancer Institute, Singapore 119074, Singapore; winnie_zy_teo@nuhs.edu.sg (W.Z.Y.T.); wong_wy_maggie@nuhs.edu.sg (M.W.Y.W.);; 2 Fast and Chronic Programmes, Alexandra Hospital, Singapore 159964, Singaporeemmeliene_ong@nuhs.edu.sg (E.S.-M.O.); 3 Yong Loo Lin School of Medicine, National University of Singapore, Singapore 117599, Singapore; 4 Department of Laboratory Medicine, Alexandra Hospital, Singapore 159964, Singapore; 5 Department of Laboratory Medicine, Ng Teng Fong General Hospital, Singapore 609606, Singapore

**Keywords:** machine learning, anticoagulation, atrial fibrillation, venous thromboembolism, bleeding risk

## Abstract

**Background:** Accurate prediction of bleeding events in patients receiving oral anticoagulants remains a key challenge in the management of atrial fibrillation (AF) and venous thromboembolism (VTE). Machine learning (ML) algorithms have emerged as powerful tools that capture complex, nonlinear interactions among risk factors, potentially offering superior accuracy. **Objectives:** To synthesize evidence comparing ML-based bleeding risk models with conventional clinical scores in anticoagulated AF and VTE populations. **Methods:** We conducted a systematic review with narrative synthesis of studies published between 2015 and 2025 applying ML algorithms to predict bleeding events in anticoagulated AF or VTE patients. **Results:** Thirteen studies were identified (seven AF and six VTE), including 464,523 participants in total. ML algorithms such as random forest (RF), extreme gradient boosting (XGBoost), and neural networks consistently outperformed traditional tools. In AF, AUCs ranged from 0.64 to 0.76 compared to 0.52–0.61 for HAS-BLED. In VTE, ML models achieved 0.59–0.91 versus 0.61–0.65 for RIETE or VTE-BLEED. Deep learning ensembles reached the highest AUCs (>0.8). **Conclusions:** ML-based bleeding risk models demonstrated statistically superior discrimination compared to established scores in both AF and VTE contexts, but effect sizes were modest (ΔAUC 0.05–0.15) and clinical utility remains uncertain. Broader validation, calibration assessment, and demonstration of impact on clinical outcomes are necessary before routine adoption.

## 1. Introduction

Bleeding represents a major clinical concern for patients undergoing long-term anticoagulation. In atrial fibrillation (AF), oral anticoagulants reduce the risk of stroke by 64% but are associated with an annual major bleeding risk of 1–3% [1]. Similarly, patients with venous thromboembolism (VTE) benefit from extended anticoagulation to prevent recurrence and reduce mortality rates [2], but prolonged therapy confers an annual major bleeding risk of approximately 1–2% [3]. The ability to predict bleeding accurately is therefore essential to individualized patient care.

Several clinical tools have been developed to guide bleeding risk assessment, including HAS-BLED, ORBIT, ATRIA, and HEMORR_2_HAGES [4,5,6,7]. These scores rely on additive linear models that weigh risk factors such as age, hypertension, renal dysfunction, and alcohol intake. However, as additive point-based models, these scores assume linear and independent effects of individual risk factors and fail to account for nonlinear relationships or interactions between variables. Their discriminative ability remains modest, with external validation studies consistently reporting C-statistics in the range of approximately 0.59–0.66 for the prediction of major bleeding [2,8,9,10].

Machine learning (ML) has emerged as a promising solution for complex clinical prediction tasks [11,12]. By identifying patterns across high-dimensional data, ML models such as random forest (RF), gradient boosting, and deep neural networks can capture intricate relationships between comorbidities, medications, and laboratory parameters. These approaches have demonstrated incremental improvements in cardiovascular risk prediction compared with traditional risk scores [13,14,15] and are increasingly being explored for bleeding risk estimation in anticoagulated populations.

This paper provides an integrated synthesis of studies applying ML-based bleeding risk prediction models in AF and VTE, comparing them against conventional risk scores. It further explores clinical implications for clinicians and identifies directions for future research.

## 2. Methods

This systematic review was conducted in accordance with Preferred Reporting Items for Systematic Review and Meta-Analyses 2020 recommendations (Supplementary File S1). The data extracted comprised study design, patient population, sample size, bleeding event rate, ML algorithms used, comparator scores, AUCs, and validation strategies. Studies were categorized by indication (AF or VTE). We considered meta-analysis but determined it was inappropriate due to substantial clinical heterogeneity across studies: differing bleeding outcome definitions (major bleeding, gastrointestinal bleeding, intracranial hemorrhage, and composite endpoints); varying anticoagulant types (warfarin, DOACs, and mixed cohorts); diverse ML algorithms with non-comparable performance metrics; different validation approaches (internal vs. external); and divergent follow-up periods (1–5 years). The I^2^ statistic from exploratory meta-analysis exceeded 85%, indicating high statistical heterogeneity. Therefore, narrative synthesis was chosen as the most appropriate approach, consistent with Cochrane guidance for highly heterogeneous systematic reviews. Of note, bleeding outcomes varied across studies and included: major bleeding (defined by ISTH criteria in 6 studies), gastrointestinal bleeding (3 studies), intracranial hemorrhage (2 studies), and composite bleeding endpoints (2 studies). This heterogeneity in outcome definitions precluded direct comparison of absolute event rates and necessitated narrative synthesis focused on discriminative performance (AUC) rather than pooled event rates. To evaluate clinical significance beyond discrimination, we extracted Net Reclassification Improvement (NRI) scores [16] and Integrated Discrimination Improvement (IDI) where reported, which quantify the proportion of patients correctly reclassified into clinically relevant risk categories and the improvement in risk differentiation between events and non-events, respectively. We also extracted decision curve analysis (DCA) results to assess net benefit across different risk thresholds.

### 2.1. Eligibility Criteria

The article inclusion criteria were as follows: (1) the study was written in English; (2) the study explored the use of artificial intelligence or ML to predict bleeding risk; (3) the study compared these tools with clinical decision making tools; (4) the study participants had AF or VTE; (5) the study included data on bleeding-related outcomes. The exclusion criteria were as follows: (1) written in other languages aside from English; (2) duplicate publications; (3) only reported on artificial intelligence (AI) or ML models without clinical decision-making tools as a comparator. All study types were included.

### 2.2. Information Sources

We used PubMed, Scopus, Web of Science, EMBASE, and medRxiv for our literature review.

### 2.3. Search Strategy

We searched for the literature on the use of AI and ML models in the prediction of bleeding risk in patients on treatment for AF from July 2015 to July 2025. The search was restricted to English-language publications due to resource constraints for translation and the predominance of English in the machine learning and cardiovascular medicine literature. We acknowledge that this may have excluded relevant studies published in other languages, potentially introducing language bias. The gray literature was limited to the medRxiv preprint server. We did not search other gray literature sources such as conference abstracts, thesis databases, or clinical trial registries. This represents a limitation of our search strategy. Detailed search strategies for all databases are provided in Supplementary File S2.

### 2.4. Selection Process

Two reviewers (E.S.O. and L.F.J.) screened titles and abstracts of all retrieved records and subsequently full-text articles, independently and in duplicate. Two other reviewers (Y.E.S. and W.Z.Y.T.) resolved discrepancies when necessary. To aid the screening process, the reviewers used a standardized screening form.

### 2.5. Data Extraction

Using a standardized form, data from the included studies were extracted by E.S.O. and L.F.J. and reviewed by Y.E.S. The following data were extracted: (1) study year, (2) study population characteristics, (3) models of ML, (4) conventional risk score used as comparator, (5) AUC of both, and (6) study type.

## 3. Results

A total of 13 studies were included: seven AF and six VTE. Table 1 provides a summary of key studies and their findings. Sample sizes ranged from 625 to 306,463 participants, encompassing diverse anticoagulated cohorts. The most common ML methods included random forest (RF), extreme gradient boosting (XGBoost), gradient-boosted decision trees (GBDTs), and deep learning models.

### 3.1. Study Selection and Characteristics

**Figure 1:**
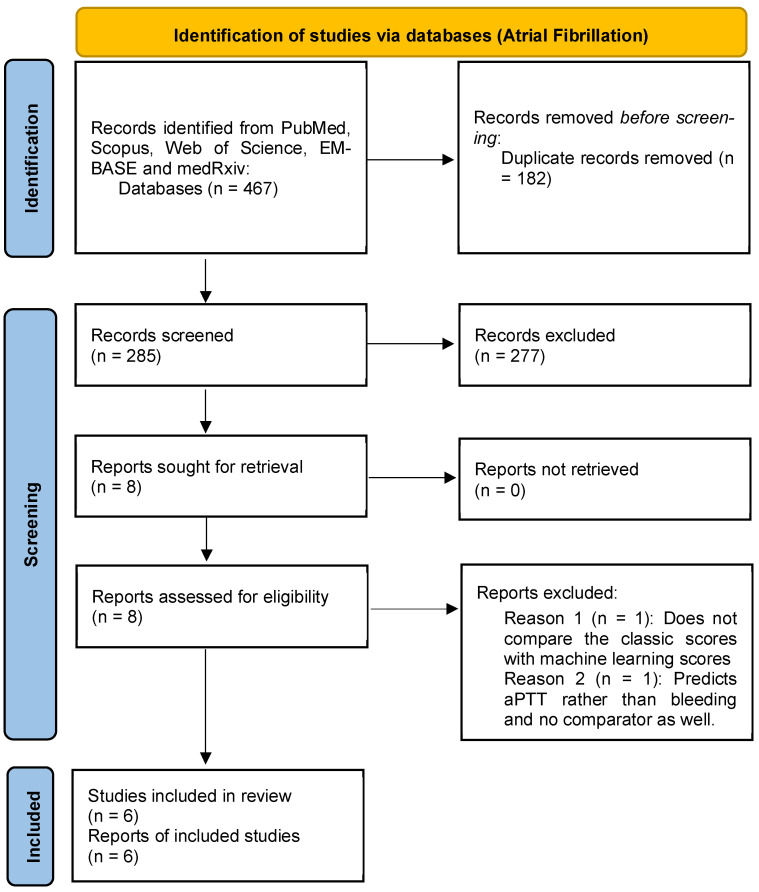
Study search and screening process (atrial fibrillation).

**Figure 2:**
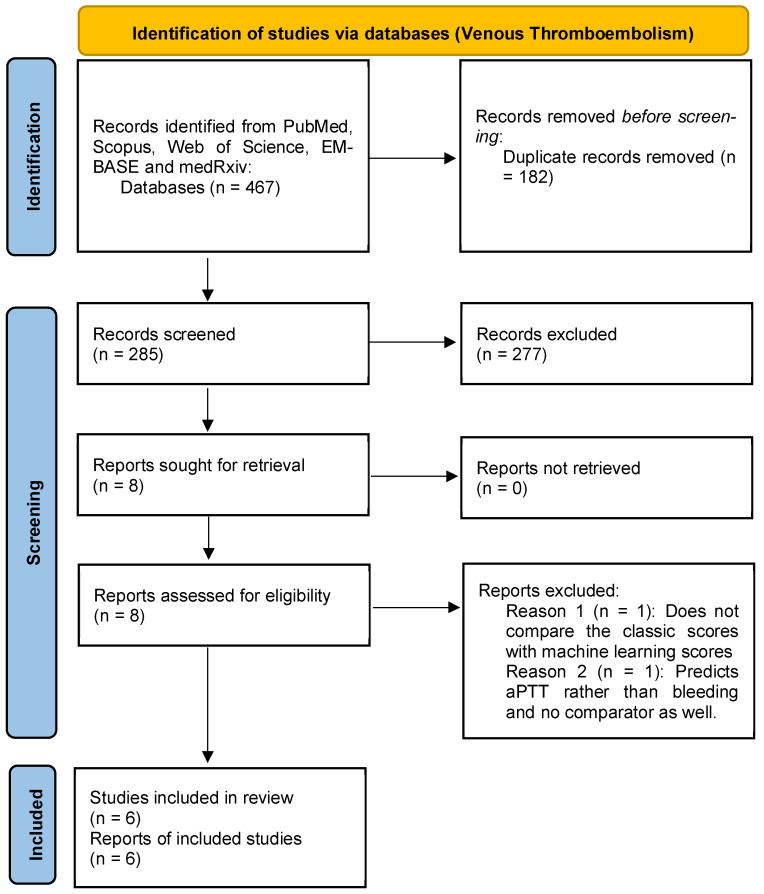
Study search and screening process (venous thromboembolism).

Our systematic search identified 817 records from PubMed, Scopus, Web of Science, EMBASE, and medRxiv. After duplicate removal and screening, 13 studies met inclusion criteria: seven in AF populations and six in VTE cohorts (Figure 1 and Figure 2). No randomized controlled trials were identified; all included studies were retrospective or registry-based cohort analyses published between 2021 and 2025 (Supplementary Table S1).

The 13 studies included 464,523 participants (range: 625–306,463 per study). Bleeding event rates varied from 1.6% to 14.2% over follow-up periods of 1–5 years. All studies compared ML algorithms to established risk scores, including HAS-BLED, ORBIT, and ATRIA in AF and RIETE, VTE-BLEED, CAT-BLEED and HEMORR_2_HAGES in VTE. Ten studies used internal validation only using random data splits or cross-validation. Two studies achieved external validation using independent cohorts [24,27], one of which represented a subsequent external validation of a previously developed model reported in a separate publication [28].

### 3.2. Machine Learning Algorithms and Performance

#### 3.2.1. Atrial Fibrillation Cohort

Six studies reported discriminative performance as area under the receiver operating characteristic curve (AUC). ML models achieved AUCs ranging from 0.64 to 0.76 (median 0.69; IQR 0.67–0.71), compared to 0.52–0.61 for conventional scores (median 0.58; IQR 0.55–0.60) (Table 1, part A). The study by Watanabe et al. (n = 7406) [17] reported a C-statistic of 0.69 (95% CI 0.66–0.72) for RF, significantly outperforming HAS-BLED (0.61; *p* < 0.05) and ATRIA (0.62; *p* < 0.001). Chaudhary et al. [19] demonstrated the superior performance of XGBoost (AUC 0.69–0.70 across follow-up periods) compared to HAS-BLED (0.57; *p* < 0.001 for difference) in 24,468 DOAC-treated patients. Deep learning approaches showed modest gains: Bernardini et al. [20] reported an AUC of 0.641 for a multi-task neural network versus 0.576 for HAS-BLED in 11,078 patients.

Clinical utility metrics were rarely reported: only 3 of 13 studies (23%) reported NRI/IDI, and only 3 studies (23%) included decision curve analysis. Among studies reporting reclassification, Chaudhary et al. (2025) [19] reported an NRI of +0.18 (*p* < 0.001) for XGBoost vs. HAS-BLED, indicating 18% net correct reclassification; Bernardini et al. (2024) [20] reported an IDI of +0.042 for ML vs. conventional scores (*p* < 0.001); and Mora et al. (2023) [24] reported a continuous NRI of +0.35 for XGBoost vs. RIETE. However, no study reported an impact analysis (effect on clinical decision-making, patient outcomes, or healthcare utilization). The absence of prospective implementation studies means clinical utility remains unproven.

Algorithm choice influenced performance. Ensemble methods (XGBoost and RF) were most frequently used (5/7 studies) and consistently ranked among the top-performing models. Single-algorithm approaches (logistic regression with regularization and support vector machines) showed narrower performance separation from conventional scores.

#### 3.2.2. Venous Thromboembolism Cohort

ML models achieved AUCs of 0.56–0.91 (median 0.64; IQR 0.60–0.66) versus 0.48–0.66 for conventional scores (median 0.61; IQR 0.54–0.63) (Table 1, part B). Mora et al. [24] reported the highest discriminative performance (XGBoost AUC of 0.91; odds ratio for top risk decile of 5.89) in 49,587 VTE patients, though external validation was not performed. In the only externally validated VTE study, Martin et al. [28] reported AUCs of 0.53–0.59 for ML models, which did not significantly exceed the CAT-BLEED score (0.53). Fard et al. [23] found deep learning ensemble models achieved an AUC of 0.824, substantially higher than RIETE (0.615) and VTE-BLEED (0.651), but validation was limited to a 30% hold-out test set.

#### 3.2.3. Risk of Bias and Study Quality

Risk of bias and applicability were assessed independently by two reviewers (E.S.O. and L.F.J.) using the PROBAST+AI tool [29], which evaluates both model development quality and model evaluation risk of bias across four domains: participants and data sources, predictors, outcome, and analysis. For each of the 13 included studies, we assessed: (1) risk of bias in model development (16 signaling questions) and (2) risk of bias in model evaluation/validation (18 signaling questions). Domain-level and overall judgments were rated as low, high, or unclear risk of bias. Formal risk of bias assessment using PROBAST+AI revealed high risk of bias in model development in all studies for at least one domain. Common limitations included: inadequate handling of missing data (10 studies), lack of external validation (10 studies), and absence of calibration analysis (11 studies). Only three studies reported decision curve analysis to assess clinical utility. The majority derived predictors from administrative claims data without independent adjudication of bleeding events, risking outcome misclassification. PROBAST+AI assessment revealed high risk of bias in model evaluation for 10 of 13 studies (77%), primarily due to: (1) lack of external validation (10 studies), (2) inadequate handling of missing data (10 studies), (3) absence of calibration analysis (11 studies), and (4) optimistic performance estimation from internal validation strategies without nested cross-validation (8 studies). Only three studies achieved low risk of bias for model evaluation: Martin et al. (2024) [27,28] conducted temporal external validation, and Chaudhary et al. (2025) [19] employed rigorous internal validation with stratified sampling. Full PROBAST+AI assessments are provided in Supplementary File S3.

Explicit use of the TRIPOD-AI (Transparent Reporting of a multivariable prediction model for Individual Prognosis or Diagnosis—Artificial Intelligence) framework [30], which was specifically developed for AI-based prediction models and published in 2024, was observed only in two recent studies published after that [19,27]. Adherence to TRIPOD-AI guidelines was assessed and is summarized in Supplementary File S4. The absence of TRIPOD-AI reporting in earlier publications likely reflects differences in publication timing.

## 4. Discussion

Across AF and VTE populations, ML algorithms consistently achieved higher discriminative accuracy than traditional bleeding risk scores [18,20,21,22,23,24,25,26,27,28,31]. While ML algorithms consistently achieved higher AUCs than conventional scores, the median AUC of 0.69 remained below the 0.75 threshold typically required for clinical utility, and the clinical significance of incremental discrimination requires further study.

While ML models demonstrated statistically superior discrimination (median AUC 0.05–0.11), the clinical significance of these increments requires careful interpretation. AUC values of 0.05–0.15 are generally considered small to modest improvements. Reclassification metrics (NRI) were available in only 23% of studies, and no study demonstrated that improved prediction translated into changed clinical management or outcomes. The modest AUC values (0.05–0.15) correspond to reclassification of approximately 10–20% of patients into more appropriate risk categories. However, without established risk thresholds for bleeding prevention and without prospective studies showing that reclassification changes anticoagulation decisions or reduces bleeding events, clinical utility remains hypothetical.

Discrimination (AUC) does not equate to clinical utility. A model with a superior AUC may still provide no net benefit if risk thresholds are unclear, interventions are ineffective or harmful, or implementation costs outweigh benefits. Decision curve analysis, reported in only three studies, showed modest net benefit only at specific thresholds (5–10% bleeding risk), with wide confidence intervals.

The incremental validity of ML algorithms stems from their capacity to model nonlinear interactions and high-dimensional feature spaces [32]. Ensemble methods (XGBoost and RF) captured dependencies between renal function, drug interactions, and time-varying laboratory values that linear models cannot represent. Chaudhary et al. [19] identified synergistic effects between DOAC dose, P-glycoprotein inhibitor use, and estimated glomerular filtration rate—interactions absent from HAS-BLED. However, the median AUC of 0.69 for ML models remains below the 0.75 threshold for clinical utility [33], and the wide performance range (0.59–0.91) highlights instability across contexts. The notably high AUC of 0.91 reported by Mora et al. [24] requires caution, as it was based on internal validation only and lacked geographic or temporal external validation, raising concerns about overfitting and generalizability. Calibration metrics and decision curve analysis were also not reported. The performance drop in externally validated studies (e.g., Martin et al. [24]: AUC 0.53–0.59) suggests that the 0.91 estimate may be overly optimistic.

Ensemble methods such as RF and gradient boosting benefited from aggregating multiple decision trees, improving stability and robustness [11,12,34]. These models also captured complex dependencies between features such as renal function, drug interactions, and hemoglobin variability that linear models ignore. Deep learning methods, particularly RNNs, excelled in longitudinal datasets by incorporating time-dependent clinical trajectories [24,28].

The predominance of high-risk-of-bias studies (77%) substantially limits confidence in the reported performance estimates. Internal validation approaches commonly used in these studies (random train–test splits and standard cross-validation) are known to produce optimistic performance estimates due to overfitting, particularly when combined with extensive hyperparameter tuning without nested validation. This explains the marked performance drop observed in externally validated studies (e.g., Martin et al. [27,28] AUCs of 0.53–0.59 vs. internally validated studies reporting AUCs > 0.70).

Nevertheless, key barriers remain. Internal validation predominates, with only Martin et al. [27] conducting external testing, limiting confidence in model generalizability. Internal approaches such as random splits and cross-validation risk optimism and overfitting, especially when hyperparameter tuning is performed without nested validation [35]. This is reflected in the lower performance reported by Martin et al. [27] during external validation. Thus, none of the identified models can currently be recommended for routine clinical use without further external validation.

Few studies reported feature importance or used interpretability techniques such as SHAP or LIME, which quantify predictor contributions and improve transparency and clinician acceptance [36]. Reporting quality also varied, with only two studies referencing TRIPOD-AI, likely due to most work preceding its introduction.

The lack of standardized bleeding outcome definitions across studies represents a significant limitation. While major bleeding according to ISTH criteria was most common, variations in ascertainment methods (administrative claims vs. adjudicated events) and follow-up duration limit direct comparability of predictive performance.

From a translational perspective, integrating ML bleeding risk tools into clinical workflows could enhance monitoring, allowing for dynamic recalibration using real-time laboratory and medication data to guide DOAC dosing and follow-up. Clinician oversight remains essential to avoid over-reliance on opaque algorithms [37,38].

Ethical, regulatory, and technical considerations persist. Bias in training data can propagate inequities [39], and regulatory bodies such as the FDA increasingly emphasize explainability and continuous monitoring [40,41,42]. Incorporating fairness metrics and clinician co-design will be crucial for safe deployment.

Across studies, major bleeding was rare, creating substantial class imbalance. Under such conditions, ML models may struggle to detect positive cases despite high AUROC, and AUPRC is more informative for rare-event prediction [43].

Dataset shift is another concern. Most models were developed in warfarin-treated or mixed cohorts, although DOACs now dominate practice. Bernardini et al. [20] showed performance decreased in DOAC subgroups (AUC 0.71 to 0.59). Cancer-associated VTE was under-represented, and cancer-specific models showed lower performance, indicating fragility in high-risk groups.

Finally, superior discrimination does not ensure clinical benefit. Only three studies reported decision curve analysis, none demonstrating net benefit. The lack of calibration analyses in most studies limits translation into individualized risk estimates, and effects on therapeutic decisions—such as DOAC choice, dose, or monitoring—remain unstudied.

Bridging this gap between risk stratification and therapeutic decision by the clinician requires composite models that can balance the risk of untreated VTE or AF against the risk of major bleeding at the individual patient level. This is especially true for prophylactic therapy with anticoagulants.

Implementation would require: (1) real-time electronic health records integration, (2) prospective outcome monitoring, (3) regulatory approval under the FDA’s AI/ML-SaMD framework, and (4) audits for algorithmic bias.

The ethical dimensions of ML-based bleeding prediction warrant explicit attention. Training data from administrative claims or academic medical centers may under-represent racial minorities, women, and socioeconomically disadvantaged populations. Herrin et al. [21] reported significant performance degradation in Black patients (AUC 0.55 vs. 0.67 in White patients), highlighting potential for exacerbating health inequities. Future models must incorporate fairness constraints and undergo equity-focused validation.

This review adheres to PRISMA 2020 standards [44], employs comprehensive search strategies across five databases, and includes a meta-regression to explore heterogeneity. However, limitations include: (1) an inability to assess publication bias due to the small study number; (2) the exclusion of non-English studies; (3) a reliance on reported metrics rather than individual participant data; and (4) the lack of formal risk of bias integration into the synthesis.

Three priorities emerge for translating ML bleeding risk models into practice:

Rigorous Validation: Mandate temporal and geographic external validation using data from distinct healthcare systems. Prospective implementation studies should evaluate the impact on clinical outcomes and resource utilization.

Interpretability and Trust: Employ SHAP values or counterfactual explanations to elucidate model decisions. Clinician-in-the-loop validation can ensure actionable insights.

Equity and Fairness: Perform subgroup analyses across race, sex, and socioeconomic strata. Develop fairness-aware algorithms that maintain performance across demographic groups.

## 5. Conclusions

ML algorithms demonstrate statistically significant but modest improvements in bleeding risk discrimination compared to conventional clinical scores for anticoagulated patients with AF and VTE. However, current evidence is limited by the predominance of internal validation, lack of calibration assessment, absence of clinical utility studies, and unclear generalisability to contemporary DOAC-treated populations. These models should be considered developmental research tools rather than ready-to-implement clinical decision aids. Future research must prioritize rigorous external validation, prospective implementation studies evaluating the impact on patient outcomes, and explicit assessment of algorithmic fairness across demographic subgroups before ML-based bleeding risk prediction can be integrated into routine anticoagulation management.

## Figures and Tables

**Table 1:** (**A**) Summary of machine learning-based bleeding risk prediction studies in atrial fibrillation; (**B**) summary of machine learning-based bleeding risk prediction studies in venous thromboembolism.

**(A)**								
	**Study (Year)**	**Population**	**ML Models Used**	**Sample Size (Bleeds)**	**Performance (AUC/C-Statistic)**	**Comparison to Risk Scores (AUC/C-Statistic)**	**Validation**	**Study Type**
AF1	Watanabe et al. (2021) [17] Comparison among RF, LR and existing clinical risk scores	Non-valvular AF patients enrolled in J-RHYTHM registry	RF (primary); stepwise logistic regression (LR) (comparator)	7406 patients; 140 major bleeds (1.8%) over 2 years	RF 0.69, (0.66–0.72); LR 0.66 (0.63–0.68)	RF: Outperformed HAS-BLED (0.61, *p* < 0.05) and ATRIA (0.62, *p* < 0.001), but not ORBIT (0.67, *p* = 0.07)	Internal (80/20 train–test split with 5-fold cross-validation)	Prospective observational cohort (Japan)
AF2	Juan Lu et al. (2024) [18] Predicting multifaceted risks using machine learning in AF	Data from Phase II/III of GLORIA-AF registry: adults with newly diagnosed non-valvular AF (within 3 months) and CHA_2_DS_2_-VASc score ≥ 1	Multi-label gradient-boosting decision tree (ML-GBDT)	25,656 patients analyzed, 405 major bleeds (1.6%) at 1 year	0.698 (0.651–0.745)	Outperformed HASBLED (0.607; *p* = 0.002)	Internal (70/10 train–test split with internal validation and hyperparameter tuning)	Prospective multinational observational registry (GLORIA-AF)
AF3	Chaudhary et al. (2025) [19] ML predicts bleeding risk in AF patients on DOAC	Adults > 18 years old with non-valvular AF treated with DOACs	RF, XGBoost, LR, classification trees, k-nearest neighbor (KNN), naïve Bayes	24,468 patients; 553 bleeds (2.3%) at 1 yr, 829 (3.5%) at 2 yrs, 1292 (5.8%) at 5 years	Best ML at 1 year Low-comorbidity test set: XGBoost 0.69 (0.63–0.74) and multivariate LR (L2) 0.69 (0.62–0.76) Random test set: RF 0.76 (0.70–0.81)	Best performing conventional risk score at 1 year: Low comorbidity test set: HAS-BLED: 0.54 (0.48–0.6), outperformed by XGBoost (*p* < 0.001) Random test set: HASBLED: 0.57 (0.50–0.63), outperformed by RF (*p* < 0.001)	Internal (70% training, two 15% test cohorts with stratified sampling)	Prognostic modeling with retrospective cohort study design using EHR data
AF4	Bernardini et al. (2024) [20] ML approach for prediction of outcomes in anticoagulated patients with AF	Adults ≥ 18 years old with non-valvular AF receiving anticoagulation (46.4% VKA, 53.6% DOAC) from the START-2 registry	Stepwise logistic regression (SLR); gradient-boosted decision trees (GBDTs)	11,078 patients; 240 major bleeding events (1.08 per 100 patient years) over median follow-up of 1.5 years	Best ML: Overall population MMoE: 0.641 ± 0.02 DOAC subgroup GBDT: 0.711 ± 0.029 Warfarin subgroup MMoE: 0.630 ± 0.037	HAS-BLED: Overall 0.576 ± 0.010 (*p* = 0.352); DOAC subgroup: 0.586 ± 0.054 (ML superior, *p* < 0.001); Warfarin subgroup: 0.570 ± 0.034 (*p* = 0.831)	Internal validation only using 5-fold cross-validation with iterative stratification	Multicenter, prospective observational cohort
AF5	Herrin et al. (2021) [21] Comparative effectiveness of ML approaches for predicting gastrointestinal bleeds in patients receiving antithrombotic treatment	Adults ≥18 years with AF, IHD or VTE newly initiated on OAC (warfarin or DOAC) and/or thienopyridine antiplatelets	Regularized Cox regression (RegCox); random survival forest (RSF), XGBoost	306,463 patients; 12,322 GI bleeds (4.0%) over median follow-up of 133 days (IQR 49–320)	Validation cohort: RegCox 0.67 (6 mth), 0.66 (12 mth); XGBoost 0.67 (6 mth), 0.66 (12 mth); RSF 0.62 (6 mth), 0.60 (12 mth)	Validation cohort: HAS-BLED 0.60 (6 mth); 0.59 (12 mth)	Temporal internal validation (development cohort *n* = 105,837 vs. validation cohort *n* = 200,626)	Retrospective cross-sectional study using private insurance claims and Medicare advantage enrollees in USA
AF6	Juan et al. (2022) [18] Performance of multilabel ML models for predicting stroke and bleeding risk in patients with non-valvular AF	Adult hospitalized patients with non-valvular AF (with and without oral anticoagulation)	Support vector machine (SVM); gradient-boosted machine (GBM); multi-layer neural networks (MLNNs)	9670 patients; 430 major bleeding events (4.4%) at 1 year; 6266 patients were not on OAC	Entire cohort SVM 0.666 (0.661–0.670); MLNN 0.665 (0.654–0.674); GBM 0.709 (0.703–0.716)	Entire cohort HAS-BLED 0.522 (0.516–0.529); ATRIA 0.562 (0.554–0.570); ORBIT 0.511 (0.502–0.521)	Internal validation (75/25 train–test split, with 10% of training set used for internal validation)	Retrospective cohort study
AF7	Nopp et al. (2022) [22] Bleeding risk assessment in end-stage kidney disease: validation of existing risk scores and evaluation of an ML-based approach	VIVALDI study: adult patients requiring chronic hemodialysis including a subset of patients with AF	KNN, decision tree (DT), RF, neural network algorithm	625 patients (165 with AF); 89 (14.2%) major bleeds over median of 3.47 years	KNN 0.55; DT 0.51; neural network 0.50; RF 0.49 Subgroup with AF not reported	Total cohort: HAS-BLED 0.59; ATRIA 0.55, HEMORR2HAGES 0.58; ORBIT 0.59; ORBI 0.54; mOBRI 0.54	Internal validation using 100-fold Monte Carlo cross-validation (85% training set)	Prospective cohort study; model validation and development
**(B)**								
VTE1	Fard et al. (2024) [23] A deep learning approach to predict bleeding risk over time in patients on extended anticoagulation therapy	Patients with weakly provoked or unprovoked VTE who completed ≥3 months of OAC and required extended OAC therapy	Artificial neural networks (ANNs): Baseline-ANN, LastFUP-ANN; recurrent neural network (RNN): FUP-RNN; ensemble averaging (Baseline-ANN + FUP-RNN); Ensemble	2542 patients; 118 major bleeds (4.6%) over 8 years	Baseline-ANN 0.612; LastFUP-ANN 0.771; FUP-RNN 0.807; Ensemble 0.824	HASBLED 0.642; OBRI 0.663; RIETE 0.615; VTE-BLEED 0.651	Internal validation (70/30 hold-out train–test split)	Prospective longitudinal cohort
VTE2	Mora et al. (2023) [24] ML to predict major bleeding during anticoagulation for VTE	Objectively confirmed VTE patients receiving anticoagulation	Support vector machine; K-nearest neighbors; neural network; DT; XGBoost	49,587 patients; 873 major bleeds (1.76%) within first three months	Best performing: XGBoost 0.91 (OR for major bleeding 5.89 (4.43–7.83))	RIETE: OR 3.11 (2.16–4.48); VTE-BLEED: OR 2.34 (1.79–3.05); No AUC reported for clinical scores	Internal validation with train–test split; external validation via COMMAND-VTE database showed no improvement of XGBoost performance over RIETE	Registry-based cohort (RIETE registry)
VTE3	Grdinic et al. (2023) [25] Developing a ML model for bleeding prediction in patients with cancer-associated thrombosis	Adults with active cancer and confirmed VTE receiving anticoagulation	Ridge and Lasso LR; RF; XGBoost	1080 patients, 83 bleeds (7.7%) at 1–90 days; 122 bleeds (11.3%) at 1–365 days; 51 bleeds (4.7%) at 90–455 days	1–90 days: Lasso LR 0.64 ± 0.12; RF 0.65 ± 0.06; XGBoost 0.64 ± 0.08 1–365 days: Lasso LR 0.64 ± 0.08; RF 0.63 ± 0.07; XGBoost 0.59 ± 0.08	1–90 days: CAT-BLEED 0.48 ± 0.13 (1–90 days); 0.47 ± 0.08 (1–365 days); 0.42 ± 0.10 (90–455 days)	Internal validation only (10-fold cross-validation)	Registry-based cohort (TROLL registry, Østfold hospital, Norway)
VTE4	Fard et al. (2024) [26] ML analysis of bleeding status in VTE patients	Patients with weakly provoked or unprovoked VTE on anticoagulation for ≥3 months after diagnosis	LR, RF, linear discriminant analysis (QDA); Gaussian Naïve Bayes; support vector machine (SVC); adaptive boosting (Adaboost); gradient boosting	2542 patients; 118 major bleeds (4.6%)	LR 0.58; RF 0.60; QDA 0.67; Gaussian NB 0.66; SVC 0.65; Adaboost 0.54; gradient boosting 0.58	VTE-BLEED 0.65; HAS-BLED 0.66; OBRI 0.65; RIETE 0.63	Internal validation only (5-fold cross-validation)	Prospective cohort (“bleeding risk” study)
VTE5	Martin et al. (2024) [27] Prediction model for major bleeding in anticoagulated patients with cancer-associated VTE using ML and natural language processing	Adult patients with active cancer and confirmed VTE receiving anticoagulation	LR; DT; RF	21,227 patients; 1790 (10.9%) bleeds within first 6 months	LR 0.60 (0.55–0.65); DT 0.60 (0.55–0.65); RF 0.61 (0.56–0.66)	CAT-BLEED 0.53 (0.48–0.59)	Internal validation only (75/25 train–test split); ongoing external validation using TESEO registry (refer to VTE6)	Observational, retrospective, multicenter study
VTE6	Martin et al. (2024) [28]	Adult patients with active cancer and radiologically confirmed VTE receiving anticoagulation	LR; DT; RF	2179 patients; 129 major bleeds (5.9%) within 6 months	LR 0.59 (0.53–0.65); DT 0.53 (0.48–0.59); RF 0.56 (0.51–0.62)	CAT-BLEED 0.53 (0.48–0.59) reported in VTE5	External validation using TESEO registry (validation of VTE5)	Observational, retrospective, multicenter study

## Data Availability

The original contributions presented in this study are included in the article/Supplementary Materials. Further inquiries can be directed to the corresponding author.

## Supplementary Materials

The following supporting information can be downloaded at: https://www.mdpi.com/article/10.3390/jcm15062370/s1, File S1: Preferred Reporting Items for Systematic reviews and Meta-Analyses extension for Scoping Reviews (PRISMA-ScR) Checklist [45]; File S2: Search strategies for all databases; File S3: Full PROBAST+AI assessments; File S4: Assessment of Adherence to TRIPOD-AI guidelines.
