# Supplementary material for: Machine Learning Models for Predicting Bleeding Risk in Anticoagulated Patients with Atrial Fibrillation and Venous Thromboembolism: A Comparative Evidence Synthesis
Source: J Clin Med. 2026 Mar 20;15(6):2370. doi: 10.3390/jcm15062370 (PMC13027241; doi:10.3390/jcm15062370)
Supplement: Supplementary file 1 [file jcm-15-02370-s001.zip › ML_Supplementary file S2.pdf]

Supplementary File S2

The following retrieval strategy was used in PubMed:

("Atrial fibrillation" OR Palpitations OR "irregular heartbeat" OR Atrial fibrillation[mesh]) AND (Anti-coagulation OR anticoagulation OR Warfarin OR Warfarin[mesh]) AND (Bleed* OR Hemorrhage OR Haemorrhage OR Hemorrhage[mesh] OR bleeding[mesh]) AND ("Artificial intelligence"[mesh] OR "Artificial intelligence" OR "machine learning" OR "precision medicine" OR "precision medicine"[mesh] OR "tree-based ensemble" OR "neural networks" OR "neural networks, computer"[mesh] OR "machine learning" OR "Clinical decision rules" OR "Clinical decision rules"[mesh])

The following retrieval strategy was used in Scopus:

(TITLE-ABS-KEY ("Atrial fibrillation" OR Palpitations OR "irregular heartbeat" AND Anti-coagulation OR anticoagulation OR Warfarin AND Bleed* OR Hemorrhage OR Haemorrhage AND "Artificial intelligence" OR "machine learning" OR "precision medicine" OR "tree-based ensemble" OR "neural networks" OR "machine learning" OR "Clinical decision rules") AND PUBYEAR > 2014) AND (LIMIT-TO (LANGUAGE , "English"))

The following retrieval strategy was used in Web of Science:

(AB=(("Atrial fibrillation" OR Palpitations OR "irregular heartbeat") AND (Anti-coagulation OR anticoagulation OR Warfarin) AND (Bleed* OR Hemorrhage OR Haemorrhage) AND ("Artificial intelligence" OR "machine learning" OR "precision medicine" OR "tree-based ensemble" OR "neural networks" OR "machine learning" OR "Clinical decision rules"))) OR (TI=(("Atrial fibrillation" OR Palpitations OR "irregular heartbeat") AND (Anti-coagulation OR anticoagulation OR Warfarin AND Bleed* OR Hemorrhage OR Haemorrhage) AND ("Artificial intelligence" OR "machine learning" OR "precision medicine" OR "tree-based ensemble" OR "neural networks" OR "machine learning" OR "Clinical decision rules")))

The following retrieval strategy was used in EMBASE:

('atrial fibrillation':ab,ti OR 'heart palpitation':ab,ti OR 'irregular heartbeat':ab,ti) AND ('anticoagulant therapy':ab,ti OR 'warfarin':ab,ti OR 'anticoagulation':ab,ti) AND 'bleeding':ab,ti AND ('artificial intelligence':ab,ti OR 'machine learning':ab,ti OR 'personalized medicine':ab,ti OR 'artificial neural network':ab,ti OR 'tree-based ensemble':ab,ti OR 'clinical decision rule':ab,ti) AND [2015-2025]/py

The following retrieval strategy was used in Medrxiv:

"atrial fibrillation" AND anticoagulation AND bleed AND "artificial intelligence"

"atrial fibrillation" AND anticoagulation AND bleed AND "machine learning"

We searched for literature on AI and ML models in the prediction of bleeding risk in patients on treatment for VTE from July 2015 to July 2025. The search was done separately from the

one for AF to include studies done in patients with VTE only. Likewise, the search was limited to those published in English.

The following retrieval strategy was used in PubMed:

("Venous thromboembolism" OR "Venous thromboembolism"[mesh] OR "deep vein thrombosis" OR "Pulmonary embolism" OR thrombus OR thrombosis OR embolism OR "venous thrombosis"[mesh] OR "pulmonary embolism"[mesh] OR thrombosis[MeSH]) AND (Anti-coagulation OR anticoagulation OR Warfarin OR Warfarin[mesh]) AND (Bleed* OR Hemorrhage OR Haemorrhage OR Hemorrhage[mesh] OR bleeding[mesh]) AND ("Artificial intelligence"[mesh] OR "Artificial intelligence" OR "machine learning" OR "precision medicine" OR "precision medicine"[mesh] OR "tree-based ensemble" OR "neural networks" OR "neural networks, computer"[mesh] OR "machine learning" OR "Clinical decision rules" OR "Clinical decision rules"[mesh])

The following retrieval strategy was used in Scopus:

((TITLE-ABS-KEY ("Venous thromboembolism" OR "deep vein thrombosis" OR "Pulmonary embolism" OR thrombus OR thrombosis OR embolism AND Anti-coagulation OR anticoagulation OR Warfarin AND Bleed* OR Hemorrhage OR Haemorrhage AND "Artificial intelligence" OR "machine learning" OR "precision medicine" OR "tree-based ensemble" OR "neural networks" OR "machine learning" OR "Clinical decision rules") AND PUBYEAR > 2014)) AND (LIMIT-TO (LANGUAGE , "English"))

The following retrieval strategy was used in Web of Science:

(AB=(("Venous thromboembolism" OR "deep vein thrombosis" OR "Pulmonary embolism") AND (Anti-coagulation OR anticoagulation OR Warfarin) AND (Bleed* OR Hemorrhage OR Haemorrhage) AND ("Artificial intelligence" OR "machine learning" OR "precision medicine" OR "tree-based ensemble" OR "neural networks" OR "machine learning" OR "Clinical decision rules"))) OR (TI=(("Venous thromboembolism" OR "deep vein thrombosis" OR "Pulmonary embolism") AND (Anti-coagulation OR anticoagulation OR Warfarin AND Bleed* OR Hemorrhage OR Haemorrhage) AND ("Artificial intelligence" OR "machine learning" OR "precision medicine" OR "tree-based ensemble" OR "neural networks" OR "machine learning" OR "Clinical decision rules")))

The following retrieval strategy was used in EMBASE:

('vein thrombosis':ab,ti OR 'venous thromboembolism':ab,ti OR 'deep vein thrombosis':ab,ti OR 'lung embolism':ab,ti) AND ('anticoagulant therapy':ab,ti OR 'warfarin':ab,ti OR 'anticoagulation':ab,ti) AND 'bleeding':ab,ti AND ('artificial intelligence':ab,ti OR 'machine learning':ab,ti OR 'personalized medicine':ab,ti OR 'artificial neural network':ab,ti OR 'tree-based ensemble':ab,ti OR 'clinical decision rule':ab,ti) AND [2015-2025]/py

The following retrieval strategy was used in Medrxiv:

· "thrombosis" AND anticoagulation AND bleed AND "artificial intelligence"

· "thrombosis" AND anticoagulation AND bleed AND "machine learning"

· "thrombosis AND "machine learning" AND anticoagulant"

· "thrombosis AND "machine learning" AND bleeding"

· "thrombosis AND "artificial intelligence" AND anticoagulant"
